# Experiences of interactive ultrasound examination among women at risk of preterm birth: a qualitative study

**DOI:** 10.1186/s12884-019-2493-2

**Published:** 2019-09-18

**Authors:** Henrika Pulliainen, Hannakaisa Niela-Vilén, Eeva Ekholm, Sari Ahlqvist-Björkroth

**Affiliations:** 1 Department of Clinical Medicine, University of Turku, Turku, Finland; 2 Department of Nursing Science, University of Turku, Turku, Finland; 3 Department of Obstetrics and Gynecology, Turku University Hospital and University of Turku, Turku, Finland; 4 Department of Psychology and Language pathology, University of Turku, Turku, Finland

**Keywords:** Interactive ultrasound, Pregnancy ultrasound, Imminent preterm birth, Psychological support

## Abstract

**Background:**

Pregnant women who are at risk of preterm birth are often stressed, anxious and depressed because of worries and fears related to the health of the unborn baby, their own health and uncertainty about the future. Only a few studies have assessed the types of psychological support that would relieve these stress symptoms among women with high-risk pregnancies. The aim of this study was to describe 1) how women at risk of preterm birth experienced an interactive 3/4-dimensional (3/4D) ultrasound examination, and 2) their need for psychological support during the antenatal period.

**Methods:**

This qualitative study was conducted at one university hospital in Finland in 2017. Women with a singleton pregnancy of 26–32 gestational weeks (gwks) were included in the study. The interactive 3/4D ultrasound included a joint observation of the baby, based on the mother’s wishes, with an obstetrician and psychologist. After the examination, the experiences were explored with a semi-structured interview. The data was analyzed using inductive thematic analysis.

**Results:**

The women enjoyed the fact that the staff were focused on her fetus and genuinely present during the session and also enabled the women to actively participate. Watching the baby and her/his activities made the baby more concrete and relieved their concerns. The need for additional psychological support varied individually.

**Conclusions:**

Interactive ultrasound examination is an interesting way to awaken mental images, increase attachment, and reduce stress. The results imply that an interactive way of jointly looking at the fetus supports pregnant women at risk of preterm birth and may be useful in clinical practice.

## Background

Pregnancy is one of the most significant transitional phases in life during which the pregnant woman goes through notable physical and emotional changes [1]. The psychological process of pregnancy has a dual focus: integrating one’s self as a parent into the current self-identity, and developing an emotional relationship with the unborn infant [2].

Pregnant women who are at risk of preterm delivery have elevated rates of depression (19–26%) and anxiety (13%) because of worries and fears related to the wellbeing of the unborn baby, their own health and uncertainty about the future [3–5]. Maternal stress during pregnancy along with other risk factors, such as tobacco use, genital tract infections, previous preterm delivery and premature contractions, increases the risk of preterm birth [6]. Depressive symptoms have an association with poor prenatal attachment to the unborn infant [7–10], which in turn predict low maternal involvement in the mother-infant interaction [11]. A high level of prenatal depressive symptoms has been shown to distort the representation of the infant and their own motherhood which, in turn, have been shown to predict dysfunctional mother-child interaction [12–14]. Further, high levels of prenatal stress have been associated with adverse obstetric and neonatal outcomes such as preterm birth (< 37 gestational weeks), intrauterine growth restriction (IUGR), and low (< 2.5 kg) birth weight at delivery [15, 16]. Results concerning the effect of hospitalization on maternal depressive and anxiety symptoms have been inconsistent, possibly due to the use of various instruments of measurement. Decrease in depressive and anxiety symptoms has been reported in several studies [4, 17], but not all [18]. However, it is not known whether psychosocial support could relieve stress symptoms in patients at risk of preterm birth.

Several studies show that ultrasound examinations have a positive impact on maternal-fetal attachment [19, 20]. In risk pregnancies, ultrasound may strengthen the maternal-fetal attachment and make the fetus more real [21]. Ultrasounds using 3/4D technique reveal clearer images and more detailed facial expressions of the fetus. It has been shown to enhance parent’s mental images of the fetus and inspire the parents to reflect on the characteristics of their baby and feel a stronger bond with the baby [22]. In line with this, de Jong-Pleij et al. (2013) [23] discovered that the effect of the ultrasound on bonding is stronger with better visibility and recognition; however, according to some reports bonding increases equally after a 2-dimensional (2D)-ultrasound in comparison with 3/4D [24]. Most studies have been done in low risk pregnancies. There are only a few studies in risk pregnancies combining psychological support with prenatal ultrasound [21, 25] in order to reduce psychological stress, anxiety and depression.

An ultrasound consultation is a method, where the fetus is observed with mother initiated interaction [21] aiming to help the parents to comprehend that the fetus is an actively exploring infant with developing senses, not just a passive recipient of different stimuli. The ultrasound consultation has been reported to increase the maternal-fetal attachment and decrease anxiety [26]. Pajulo et al. (2016) [25] further developed this method by using 3/4D ultrasound and adding an interactive mentalization focus, as an intervention for pregnant women with substance abuse problems.

The aim of this study was to explore the experiences of women at risk of preterm birth of an interactive ultrasound and the applicability of an interactive ultrasound as antenatal support. In addition, pregnant women’s need for psychological support during hospitalization was examined.

## Methods

### Design and setting

This qualitative descriptive pilot study was conducted at a University hospital in Finland between April and December 2017. The hospital is a tertiary level hospital covering approximately 4000 births annually. Women at risk of preterm birth are treated on a hospital ward accommodating 22 beds by an obstetric team including maternal-fetal medicine specialists; the mothers have the possibility of a discuss with a neonatologist and can visit the neonatal intensive care unit (NICU) before delivery.

### Sample and data collection

A convenience sample of pregnant women who were hospitalized because of a risk of preterm birth were recruited into the study. The pregnant women were eligible if: 1) they had singleton pregnancy, 2) with gestational weeks (gwks) between 26 and 32, 3) were fluent in Finnish and 4) aged over 18 years. Women with breaking of water and body mass index over 30 were excluded. Availability of 3/4D-ultrasound device had an impact on patient selection, because interactive ultrasound examinations were done in prearranged times. None of the approached women refused to participate. The women were informed and recruited no earlier than on the second day of their hospitalization. The women participated in an interactive 3/4D-ultrasound examination, which included a joint observation of the baby with the pregnant woman, a specialist in maternal fetal medicine and a psychologist specialized in infant mental health. The session lasted about 40 min and progressed through the certain phases (Table 1.). The focus of the session was to observe the fetus together according to the mothers’ wishes. Her thoughts and experiences were listened to and discussed while watching the fetus. 2D-ultrasound was alternately used during the session, because certain fetal actions, for example swallowing, are more clearly seen in a 2D view.

**Table 1:** Content of the interactive ultrasound examination

1. Starting phase	
– Asking how the day has been for the mother and the baby	
– Asking when the mother has seen the baby last time	
– Asking mother’s wishes according to the session: Is there something you want to look at?	
2. The joint observation phase	
– The obstetrician explains what is presented on the ultrasound screen	
– Observing together baby’s movements, swallowing, sucking, licking …	
– Professionals are keeping their comments on a behavioral level, leaving space for the mother to create own meanings	
– Sometimes mother can be asked to reflect on baby’s experiences or feelings	
– Sometimes mother can be asked to reflect on own feelings while watching the baby	
– If mother is expressing a worry that is always listened to and reflected together and answered, if possible	
– Mothers positive observations are shared	
3. Closing up phase	
– Is there something else you would like to look at?	
– How has it been for you to observe your baby like this?	
– The obstetrician explains without medical terms her impression about baby’s well-being	

Afterwards, the women were asked about their experiences of the ultrasound session and their potential need for psychological support in a semi-structured interview (Table 2). The psychologist conducted the interviews immediately after the ultrasound examination in all cases except one interview that was done the following day. The interviews were audiotaped.

**Table 2:** Interview for the mothers

Interview for the mothers The interviewer revises the meaning of the interview shortly and checks the health and the willingness of the woman at the moment.	
1. Experience of the interactive ultrasound examination How did you experience the interactive ultrasound examination? Did it differ from your previous experiences of ultrasound examinations? How did it differ? What did you think about the examination room and how we were settled there? What kind of thoughts did you have during ultrasound examination? What did you think about your baby? How did it feel to look at your baby and his/her movements together with the obstetrician? • Feelings towards the baby? • Feelings about your parenthood? • Feeling about the attachment towards the baby? What kind of emotions did the interactive ultrasound examination awake in you? • positive/negative emotions? Did your observations change the mental image or thoughts about the baby? If you were offered a new opportunity to participate in interactive ultrasound examination, would you take part? Did you miss something or was there something that could have been done differently? Did you experience this useful or supportive? If yes, how?	
2. Experience about emotional support during this pregnancy What kind of mood have you been lately? Have you longed for emotional support las days? Do you feel like you have got the kind of emotional support lately, that you needed? • If yes, what kind of support? • If no, what kind of support you would have needed? If support was offered from the prenatal ward regularly to the families, what kind of support would it be? What would be the best option for you: psychologist’s appointments, peer support groups, interactive ultrasound examinations, learning videos? Have you met neonatologist? How did you experience it?	
3. Did the interactive ultrasound examination offer enough support? (This question can be skipped if the interviewee has previously answered that interactive ultrasound examination was useless) Suggestions for improvement if the interviewee experiences the session useful	
4. What would be good psychological support during pregnancy in your case?	

### Ethical considerations

The study was approved by the Ethics Committee of the Hospital District of Southwest Finland, case number 95/1801/2017. Written informed consent was obtained from the participants.

### Data analysis

The audiotape data were transcribed verbatim and analyzed using inductive thematic analysis [27], which enables the identification, analysis and reporting of meaningful themes from the data. After several rounds of re-reading, the data were systematically coded by H.P. using NVivo11 software. Similar codes were collated and initial themes were formed such as ‘mother as a valued participant’, ‘baby as an individual’, ‘more concrete mental image of the baby’ and subthemes such as ‘hospitalization was experienced as a stressful situation’, ‘pleasant 3/4D ultrasound examination’. H. N-V. familiarized herself with the raw data and confirmed the initial analysis. The researchers together formed a comprehensive understanding about the underlying patterns in the data. In the last phase of the analysis, the themes were defined and named. The analysis was performed with the original Finnish data and the direct quotes were translated in English.

## Results

A total of twelve pregnant women participated in the study (Table 3). Their median age was 28 years (range 22–31) and pregnancy duration 30 + 2 (range 28 + 0–32 + 3) gwks and eight of the participants were primiparas. The women’s experiences of the interactive ultrasound examination were compiled under three main themes: 1) Pregnant woman as an active and equivalent participant, 2) The fetus as a real person and 3) Strengthened mental image of the baby.

**Table 3:** Background characteristics

	Mean	Range	Median
Age (years)	28	22–31	28.5
Gestational weeks at 3/4D ultrasound examination	30 + 2	28 + 0–32 + 3	30 + 2.5
Ward time (days)	10	2–21	4
Gestational weeks at birth	36 + 3	31 + 6–39 + 3,	37 + 3

### Pregnant woman as an active and equivalent participant

Women experienced the atmosphere as peaceful and open and that the obstetrician and the psychologist were interested in her and her fetus. Most of the women described hospital admission as hectic, unexpected and confusing with the uncertainty about the situation and the baby’s condition creating stress and anxiety. The women related, that they did not dare to ask the doctor and midwife about their worries because they were confused and did not want to interrupt the staff’s work. Some of the women did not even know why they were hospitalized. The interactive ultrasound session was therefore experienced as pleasant and providing an opportunity to ask questions. The women felt they were treated as appreciated participants whose thoughts and questions were valuable and important.

Many women described their previous ultrasound examinations as professional-centered events. They experienced that during regular ultrasounds the sonographer only focused on measuring the fetus and the assisting person on entering the measurements into electronic patient files. The professionals led the situation and discussed with each other while the women felt they were placed in a minor role. Contrary to their previous experiences, the women described the interactive ultrasound session as easy-going, peaceful and relaxed, and the experience as positive. The women enjoyed the interactive atmosphere during the session and the obstetrician’s and psychologist’s ability to take part in the conversation. The women felt that the sonographer and psychologist were truly present during the session and really focused on the fetus and her. The women appreciated the fact that the obstetrician considered their wishes on what they wanted to see in the ultrasound. The feeling of presence and that answers were given in simplified language avoiding medical terms, encouraged the women to ask questions freely. According to the women’s experiences, open discussion during the ultrasound session offered different perspectives in a secure way.

*I found it [ultrasound examination] nice. Usually in my previous ultrasounds, there has been a doctor and a nurse and it has been like they have been talking to each other. And there have been a lot of numbers, the doctor has listed the measures and the nurse has recorded them. I have felt like being in a minor role, only lying there. This ultrasound session was more interactive. I felt like being a part of it and I was told what is going on.* (31+0 gwks, primipara)

### The fetus as a real person

The pregnant women enjoyed the ultrasound experience because the fetus was considered as a real person, a subject, not just an object to be measured. The women’s previous ultrasound examinations were usually conducted as two-dimensional. The 3/4D ultrasound technique offered a unique opportunity for the women to see their babies from a different perspective. Seeing and focusing on the baby, not only a two-dimensional image to be measured, generated positive emotions among the women. During the interactive ultrasound session, the women appreciated that their fetus was called and seen as a baby or ‘your baby’. The women enjoyed peacefully observing details and activities of the baby and were happy to discuss them with the obstetrician and the psychologist. The mothers were positively surprised about the babies’ activities, as they saw a lot more happening inside the womb than they were able to feel. With 3/4D-technique, the women were able to see their baby’s face and facial expressions as well as activities such as swallowing amniotic fluid and licking for example, the uterine wall or placenta. Movements of the upper limbs were also often seen such as putting the hands on the face, sucking the thumb and playing with the umbilical cord. Based on the observation, the women started to interpret their babies’ facial expressions and behavior and described, for example, that the baby looked happy and satisfied.

*It was very nice, especially when I could see for a first time that the baby was sucking his thumb and everything else that I have never been able to see in my previous ultrasound examinations. So comparing with my previous experiences this was really fun and nice and different session, and I had high spirit because of that.* (28+0 gwks, primipara)

*I think it was really nice and we spent quite a long time chatting about what he was doing with the umbilical cord and he looked like smiling a bit and being like happy. He clearly looked satisfied with being there and I enjoyed seeing him moving around. I had very warm feelings toward him and I think he is fine there.* (30+1 gwks, primipara)

### Strengthened mental image of the baby

During the ultrasound session, the baby became more concrete, as the mothers could see the baby’s face and activities. The women experienced the fetus as complete. They were rather surprised that their fetus looked like a real human being. Seeing the baby during the session awakened emotions, such as closeness and a deeper attachment. Further, it confirmed the mothers that despite the risk of preterm birth the baby seemed to be well inside the womb. Based on these observations some women illogically concluded that their babies were not going to be born yet. However, the women were emotionally relieved after the interactive ultrasound session.

*I became sensitive; this session strengthened the relationship between me and my baby. I have been thinking about him and when I could simultaneously see him and feel his movements, it really strengthened our relationship although it has already been strong. However, the visual perception and physical touch at the same time…* (31+3 gwks, primipara)

### Suggestions for additional emotional support

All the participating women appreciated the interactive ultrasound session as a good method for emotional support during hospitalization due to an imminent birth. Watching the baby relieved their worries. According to the participants, this method would be useful in clinical practice. Many of the women mentioned feeling safe while being hospitalized; they trusted the hospital system and valued the midwives and doctors. However, all the women emphasized that it would beneficial if professional emotional support was available on the ward and could be provided based on individual needs. If the woman had a strong supportive network of family and friends, the need for additional emotional support was low. All the women valued peer support from the other women on the ward or from social media.

## Discussion

We found that pregnant women with an imminent preterm birth experienced the interactive ultrasound as supporting their active and equivalent participation which also enabled them to consider the fetus as a real person and strengthen the mental image of the baby. Any need for additional emotional support depended on the woman’s social network and should, therefore, be provided on an individual basis. The interviews revealed the women’s appreciation of their thoughts and questions being valued during the ultrasound sessions. Most importantly, they experienced that they were able to participate actively and equally in contrast to their previous ultrasound examinations, which they described as professional-centered. Professional-centered care may lead to a passive role during the ultrasound examination and subsequent decision-making. Allowing and encouraging the pregnant women to participate actively in the observation of the fetus may have empowered them to present questions and their concerns. Our finding is in line with previous results showing that the responses of pregnant women to the ultrasound examination is more favorable when it is performed in an interactive way [26]. Because the women were active participants and their babies were seen as real persons, the women’s mental images of their fetuses were strengthened.

The maternity care in Finland strives to be patient-centered. Thus, it was surprising that some of the women had not understood the reason for their hospitalization and had not dared to ask the hospital staff about it. This implies that informing the patient about decisions based on any medical examination should be done explicitly without using professional terms and by involving the patients in the decision-making. Active participation has been related to increased empowerment and dignity and the loss of powerlessness in family-centered care [28]. Early interventions activating parents’ participation in neonatal setting have been shown to facilitate parenting and parental well-being [29, 30] as well as the development of the preterm infants [31]. It is possible that active participation in prenatal treatment has similar effects, as the women reported that seeing the baby during the ultrasound aroused emotions, such as feelings of closeness and deeper attachment. The first trimester ultrasound screenings already confirm the forthcoming parenthood [32, 33].

The women appreciated that the baby was seen as a person during the interactive ultrasound examination. Indeed, most women (92%) experience the fetus as a separate individual during the third trimester of pregnancy, and some already during the second (63%) or even the first (30%) trimester [34]. Between the fourth and seventh month of pregnancy women also create rich and specific mental representations of the baby [2] that are related to a stronger emotional bond to the unborn baby. This progress in mental representations is initiated by the moment when a woman senses the movements of the baby. Because all the participating women were between 28 and 32 gestational weeks, they were likely to imagine the baby as a real person and an individual. The women reported, that seeing the 3/4D view of the baby and observing the baby’s behavior in the womb made the baby more concrete and real for them. Previously, women’s representations of the unborn baby have been shown to be complementary to their representations of their own care giving role [35]. For example, if a woman imagines herself as a loving and caring mother, she imagines the baby as calm and happy. With ultrasound the representations related to the baby are enriched by observation of the real infant and slowly separated from the representations related to their care giving role [1, 2]. Importantly, the mothers also reported that the session awakened positive emotions, such as closeness and a deeper attachment, towards the baby. Interactive ultrasound is a unique way to approach pregnant women. It has not previously been applied to pregnant women with a risk of preterm birth. The women’s overall experience of 3/4D-ultrasound examination as positive in the present study is in line with previous findings [19, 22–24, 36–38]. Most of the previous studies regarding the impact of 3/4D-ultrasound on maternal-fetal bonding are relatively outdated and the method evaluating the bonding varied. However, 3/4D ultrasound has been shown to improve maternal fetal-bonding in the third trimester [23] and in first and second trimesters as well [19, 24, 36, 38]. Due to advanced technology, the quality of the 3/4D-images have improved and thus enables more realistic images of the fetus. The joint observation probably intensifies the effect of the 3/4D ultrasound image as regards maternal bonding to the infant.

Maternal prenatal attachment predicts the postnatal mother-infant interaction [11]. A low level of attachment is associated with negative emotions, depression and weak support from the family and partner [14, 39]. In preterm delivery, bonding to the infant is at risk because of early interruption of the psychological preparation for parenthood [40, 41]. The psychological influence of 3/4D ultrasound should be further investigated in risk groups [19, 22, 24, 25, 36] because there is increasing evidence that prenatal distress may be detrimental to the cognitive, psychomotor and behavioral development of the infant [42]. Thus, active and non-pharmacological prenatal interventions are needed.

An interactive ultrasound requires some training and personnel resources, but is feasible in clinical setting [25]. The presence of both the maternal fetal specialist and the psychologist enabled them to give full input. While the psychologist concentrated on joint observations with the woman, the obstetrician had the freedom to focus on the ultrasound view of the fetus. This study highlights the importance of communication between health care professionals and pregnant women as regards the fetus during an ultrasound examination. However, it is difficult to determine whether the advantages of the interactive ultrasound examination are due to improved communication or the method itself. Thus, the core issues of the intervention, listening to the mother, personalizing the fetus and giving emotional support, can easily be reached. Even though 3/4D- images give more realistic view of the fetus, this method may also be applied within the 2D-technique. Looking at fetus’ actions during routine ultrasound examinations with better communication and treating the pregnant woman as an active participant may strengthen the mental images in daily clinical setting.

It is also important to consider potential disadvantages of the interactive ultrasound method. The image of the 3/4D ultrasound may affect negatively on the pregnant woman’s developing mental images about her baby, but there is not enough knowledge available about diverse effects on the 3/4D-images on parental representations. However, the risk of negative impact is probably smaller when the image is properly explained and explored together with a parent. A strengthened emotional bond to the unborn infant after interactive ultrasound could also be considered a potential risk in the case of a woman losing the fetus or newborn infant. However, the literature shows [20, 43] that mothers’ well-being after a neonatal death is associated with their satisfaction with the support they received from the hospital staff after the death of the infant and the opportunities to create special and concrete memories of their baby. The interactive ultrasound can be seen as an opportunity for a pregnant woman to create memories of the unborn infant.

The small sample size is a limitation, although data saturation, indicated by repetitive responses in the interview was reached. In addition, the data provided a coherent understanding of the topic. The interviews were conducted based on a pre-designed interview guide which confirmed the dependability. The interviews were performed by the psychologist who was present at the ultrasound sessions, and this might have affected the participants’ responses. The transferability of the results may be limited. The aim of this pilot study was to map the women’s experiences of the interactive use of a 3/4-ultrasound in a group of women at risk of preterm birth. The results provide promising information about the interactive use of the ultrasound and encouragement to continue studying the topic.

## Conclusions

The results of the qualitative pilot study imply that an interactive way of looking at the fetus during an ultrasound examination supports pregnant women at risk of preterm birth. It is a potentially new way to awaken maternal mental images, and thus increase maternal attachment to the infant as well as reducing worry during pregnancy. The method is relatively easy to implement into public healthcare and thus, may be a promising clinical practice in treatment of women with risk pregnancy. However, the effectiveness of this method needs rigorous further evaluation.

## Data Availability

The datasets generated and analyzed during the current study are not publicly available due to obligation to maintain confidentiality but are available from the corresponding author on reasonable request.

## Abbreviations

2D: 2-dimensional
Gwks: Gestational weeks
IUGR: Intrauterine Growth Restriction
NICU: Neonatal IntensiveCcare Unit
3/4D: 3/4-dimensional
